# Diet for the Sick

**Published:** 1893-06

**Authors:** 


### Book Reviews.

DIET FOR THE SICK. by Miss E. Hibbard, Principal of Nurses Training School Grace Hospital, Detroit, and Mrs. Emma Drant, Matron of Michigan College of Medicine Hospital, Detroit; to which has been added Complete Diet Tables for various diseases and conditions, as given by the highest authorities. Detroit, Mich., The Illustrated Medical Journal Co., Publishers. Paper, 74 pages. Price, postpaid, 25 cents; 6 for $1.00.

This little book is a worthy supplement to any cook book, as it deals only with the dishes suitable for the sick and convalescent; the receipts being favorite ones in use daily in the hospitals wherein the authors are employed. To this has been added the various authorized Diet Tables for use in Anæmia, Bright's Disease, Calculus, Cancer, Chlorosis, Cholera Infantum, Constipation, Consumption, Diabetes, Diarrhœa, Dyspepsia, Fevers, Gout, Nervous Affections, Obesity, Phthisis, Rheumatism and Uterine Fibroids. It also gives various nutritive enemas. The Physician can use it to advantage in explaining his orders for suitable dishes for his patient, leaving the book with the nurse.

